# Decline of Psychological Health Following the Designation of COVID-19 as a Pandemic: Descriptive Study

**DOI:** 10.2196/24964

**Published:** 2021-04-22

**Authors:** Darpan I Patel, Yazmin Gamez, Lajja Shah, Jaini Patel

**Affiliations:** 1 Biobehavioral Research Laboratory School of Nursing University of Texas Health Science Center at San Antonio San Antonio, TX United States; 2 Department of Rehabilitation & Sports Medicine Sir H N Reliance Foundation Hospital Mumbai India

**Keywords:** anxiety, COVID-19, descriptive study, mental health, pandemic, physical health, quality of life, stress

## Abstract

**Background:**

COVID-19 was declared a pandemic by the World Health Organization on March 11, 2020, and as of this writing, Texas, United States, has reported >675,000 cases with over 14,000 deaths. Many of the preventive measures implemented during the pandemic can increase sedentary lifestyles, which can lead to the development of chronic diseases, including obesity, among the general population and cause serious threats to people’s physical health and overall quality of life. Individuals with pre-existing comorbidities are at an increased risk of COVID-19 and may hence have higher levels of stress.

**Objective:**

This study aimed to investigate the relationship between physical activity levels and mental health status on an individual level and to compare them between those with and those without comorbidities in a cohort of Texas residents, before and after COVID-19 was declared a pandemic.

**Methods:**

An electronic survey was disseminated throughout various regions of Texas. In total, 160 individuals were asked questions about their demographic characteristics, time spent on daily physical activities, and daily mental health status before and after COVID-19 was declared a pandemic. Frequency distributions and descriptive statistics were analyzed.

**Results:**

Overall, 94 (58%) participants reported having ≥1 medical condition, and 31 (13.1%) had >3 medical conditions. Physical activity levels among participants with ≥1 pre-existing comorbidity drastically—but not significantly—decreased, as evident from a 10% increase in sedentary lifestyles after COVID-19 was declared a pandemic. On the contrary, we observed a 9% increase in the number of individuals without a pre-existing comorbidity who reported 30-60 min of physical activity per week. There was a 2-fold increase in the number of participants reporting more frequent feelings of nervousness, too much worry, trouble relaxing, and the fear of something awful happening after the pandemic. More specifically, individuals with pre-existing medical conditions reported, on average, a 10% higher incidence of feelings of stress, anxiety, and sadness compared to their healthy counterparts after COVID-19 was declared a pandemic.

**Conclusions:**

Stressful life conditions and chronic comorbidities are risk factors that can affect mental health and reduce the ability to perform activities of daily life. Therefore, when implementing pandemic protocols, municipalities should consider providing mental health support to their citizens to protect them from this rather inconspicuous adverse effect.

## Introduction

COVID-19 was declared a pandemic by the World Health Organization on March 11, 2020 [1], and Texas has reported >675,000 cases and 14,000 deaths as of this writing. The routines of individuals have been impacted since the beginning of the pandemic owing to the implementation of restrictions and preventative measures such as social distancing, self-isolation, stay-at-home orders, and closure of businesses and recreational facilities. Many of these protocols can have a negative effect, promoting a sedentary lifestyle and decreasing physical activity levels, which can lead to the development or exacerbation of chronic diseases and mental health issues [2-5]. This study aimed to explore the relationship between physical activity levels and mental health status at an individual level in a cohort of Texas residents before and after COVID-19 was declared a pandemic. We hypothesize that the declaration of COVID-19 as a pandemic, which led to the implementation of social distancing and self-isolation protocols, would negatively impact mental health, increase stress and anxiety, and decrease physical activity levels among all study participants.

## Methods

### Recruitment

Texas residents aged ≥18 years were recruited through social media and email, using convenience and snowball sampling over a 2-week period in July 2020. Respondent identities were anonymized, and the study was exempt from ethics review for the use of human subjects by the institutional review board of the University of Texas Health Science Center at San Antonio.

### Survey Instrument

A shortened survey instrument initially developed by Flanagan et al [6] was used to capture respondent demographics, medical history, and physical and mental health status. Questions related to physical and mental health were constructed in a manner to determine health status before and after the implementation of COVID-19 quarantine protocols. Study data were collected and managed using Research Electronic Data Capture (REDCap) [7] tools hosted at the University of Texas Health Science Center at San Antonio. REDCap is a secure web-based software platform designed to support data capture for research studies, providing (1) an intuitive interface for validated data capture, (2) audit trails for tracking data manipulation and export procedures, (3) automated export procedures for seamless data downloads to common statistical packages, and (4) procedures for data integration and interoperability with external sources.

#### Demographics and Medical History

Data on the participants’ gender (male, female, or no response), age, marital status, employment status, household number, ethnicity, and race were collected. Participants were asked to indicate whether they have been diagnosed with any chronic medical conditions, including cardiovascular, respiratory, gastrointestinal, genitourinary, kidney, hematologic, infectious, dermatological, ophthalmologic, endocrine, musculoskeletal, oncologic, or neurologic disease. The demographic characteristics of the survey participants are presented in Table 1.

**Table 1:** Demographic characteristics of the survey participants (N=160).

Characteristic		Number of participants, n (%)
**Race**		
	American Indian or Alaskan native	0 (0)
	Asian	10 (6.3)
	Black or African American	3 (1.9)
	Native or Pacific Islander	3 (1.9)
	White	134 (83.8)
	Not reported	8 (5.0)
	Unknown	2 (1.3)
**Ethnicity**		
	Hispanic or Latino	65 (40.9)
	Not Hispanic or Latino	86 (53.9)
	Not reported	7 (4.5)
	Unknown	2 (1.3)
**Gender**		
	Female	138 (86.3)
	Male	20 (12.5)
	Not reported	2 (1.3)
**Age (years)**		
	18-25	17 (10.9)
	26-35	44 (27.6)
	36-45	30 (18.6)
	46-55	34 (21.2)
	56-65	28 (17.3)
	66-75	7 (4.4)
**Pre-existing diagnosed medical conditions (n=94; percentages reported considering a sample of 160 participants)**		
	1	52 (32.5)
	2	21 (13.1)
	3	13 (8.1)
	≥4	8 (5.0)

#### Questions on Physical Activity

Participants were asked to provide details regarding their physical activity levels before and after COVID-19 was declared a pandemic. Specifically, they were asked to provide details regarding their physical activity or exercise minutes per day. Participants were provided the following categorical variables as choices: 0-30 minutes, 30-60 minutes, 60-90 minutes, 90-120 minutes, or >120 minutes of daily physical activity.

#### Questions Related to Psychological Status and Mental Health

Participants were asked if they were worried about their physical health with respect to the COVID-19 pandemic. They were also asked about generalized stress, anxiety, and sadness before and after the COVID-19 pandemic. Furthermore, 8 questions were related to the participants (1) feeling nervous, anxious, or on edge; (2) not being able to stop of control worry; (3) worrying too much about different things; (4) having trouble relaxing; (5) being restless and finding it difficult to sit still; (6) becoming easily annoyed or irritable; (7) feeling afraid that something awful might happen; and (8) having difficulty getting things done because of these mental health issues. Participants selected either (1) not at all, (2) several days, (3) over half the days, or (4) nearly every day. Each response was converted to a numerical score for analysis.

### Statistical Analysis

Descriptive statistics were calculated to summarize the response frequency. Respondents who declined to answer a question were considered missing and excluded from the calculated proportions. We report all summary statistics. For paired testing of questions that asked for pre– and post–COVID-19 comparisons, we used a paired samples *t* test to analyze mean values. The significance level was set at Cronbach *α*=.05, and all tests were 2-sided. All data were processed using SPSS for Windows (version 23.0, SPSS Inc).

## Results

### Participant Demographics

In total, 160 individuals responded to the social media posts. The vast majority of respondents were married, white, non-Hispanic or -Latino females. Overall, 65 (41%) respondents identified themselves as Hispanic or Latino (Table 1), which was more than the 31% estimated census of Hispanic or Latinos in Texas. More than half of the respondents (n=91, 57%) were aged <45 years and had full-time jobs (n=117, 73%), while 30 (19%) indicated that they were unemployed.

Furthermore, 94 (58%) participants reported having ≥1 pre-existing medical condition, and 21 (13.6%) respondents had >3 pre-existing medical conditions. Medical conditions with the highest frequency included hypertension (22.3% of all responses), respiratory problems (14.0%), endocrine disorders (13.4%), and gastrointestinal problems (12.7%). Participant responses to questions on pre-existing medical conditions are presented in Table 2.

**Table 2:** Distribution of the responses of participants with pre-existing medical conditions (N=160).

Pre-existing medical condition	Number of responses, n (%)^a^
Cardiac or heart disease	6 (3.8)
Respiratory problems	22 (14.0)
Gastrointestinal problems	20 (12.7)
Genitourinary or kidney disease	5 (3.2)
Hematologic condition	10 (6.4)
Infectious disease	1 (0.6)
Dermatologic condition	13 (8.3)
Ophthalmologic condition	7 (4.5)
Endocrine conditions	21 (13.4)
Diabetes	10 (6.4)
Musculoskeletal conditions	8 (5.1)
Hypertension	35 (22.3)
Cancer	6 (3.8)
Neurologic or psychiatric condition	14 (8.9)

^a^Among 160 respondents, 94 reported having ≥1 pre-existing medical condition. Individuals were asked to mark all medical conditions they have been diagnosed with, allowing them to select >1 response. Percentages are in relation to the total sample of 160 survey responses.

### Slight Reduction in Physical Activity in Response to the Declaration of COVID-19 as a Pandemic

No significant differences in self-reported physical activity were observed for the entire cohort of respondents. Similarly, no significant differences were observed in the subgroups of individuals with (*P*=.81) or without (*P*=.91) pre-existing medical conditions. Physical activity was slightly—but not significantly (*P*=.81)—reduced among participants with ≥1 pre-existing condition, as evident from a 10% increase in sedentary lifestyles after COVID-19 was declared a pandemic. On the contrary, we observed a 9% increase in the number of individuals without a pre-existing conditions reporting 30-60 min of physical activity per week.

### Negative Effect of the COVID-19 Pandemic on Psychological Health

Respondents were asked how the quarantine protocols in Texas impacted their psychological health (Table 3). We observed significant increases in feelings of fear, annoyance, restlessness, worry, nervousness, sadness, anxiety, and stress (*P*<.001) among the survey respondents. Specifically, participants reported more frequently feeling nervous, worrying too much, having trouble relaxing, and feeling afraid something awful might happen after the pandemic. This negative impact on psychological well-being interfered with their ability to accomplish their work, tasks, or interact with other people. Similar results were obtained among individuals with and those without pre-existing medical conditions. The only exception is that individuals without pre-existing medical conditions reported that the COVID-19 pandemic had no significant impact on their ability to relax (*P*=.08).

**Table 3:** Changes in psychological health resulting from the COVID-19 pandemic.

Items	All respondents			Respondents with pre-existing medical conditions				Respondents without pre-existing medical conditions		
	Pre–COVID-19, mean (SD)	Post–COVID-19^a^, mean (SD)	*P* value	Pre–COVID-19, mean (SD)	Post–COVID-19, mean (SD)	*P* value	Pre–COVID-19, mean (SD)		Post–COVID-19, mean (SD)	*P* value
Feeling nervous, anxious, or on edge	1.78 (0.77)^b^	2.36 (0.91)	<.001	1.88 (0.77)	2.53 (0.98)	<.001	1.63 (0.75)		2.13 (0.74)	<.001
Not being able to stop or control your worry	1.57 (0.82)	1.95 (0.93)	<.001	1.63 (0.84)	2.04 (1.03)	<.001	1.41 (0.80)		1.82 (0.77)	.003
Worrying too much about different things	1.73 (0.81)	2.31 (1.02)	<.001	1.78 (0.75)	2.40 (1.06)	<.001	1.68 (0.90)		2.18 (0.98)	<.001
Trouble relaxing	1.70 (0.89)	2.09 (1.03)	<.001	1.81 (0.91)	2.29 (1.06)	<.001	1.58 (0.86)		1.84 (0.95)	0.08
Being so restless that it is difficult to sit still	1.27 (0.62)	1.55 (0.81)	<.001	1.31 (0.63)	1.64 (0.91)	.002	1.21 (0.62)		1.45 (0.65)	.05
Becoming easily annoyed or irritable	1.68 (0.67)	2.07 (0.86)	<.001	1.70 (0.68)	2.16 (0.88)	<.001	1.68 (0.66)		2.00 (0.81)	.01
Feeling afraid as if something awful might happen	1.41 (0.70)	2.04 (0.99)	<.001	1.45 (0.73)	2.12 (1.01)	<.001	1.38 (0.68)		1.92 (1.01)	<.001
How difficult did these factors make it for you to do your work, take care of things, or get along with other people	1.22 (0.73)	1.69 (0.86)	<.001	1.34 (0.92)	1.77 (0.95)	<.001	1.19 (0.66)		1.62 (0.72)	<.001

^a^After COVID-19 was declared a pandemic by the world Health Organization on March 11, 2020.

^b^Participants selected either of the following responses: (1) not at all, (2) several days, (3) over half the days, or (4) nearly every day. Each response was converted to a numerical score for analysis. For the question on difficulty level, participants selected either of the following responses: (0) I did not experience any of the conditions, (1) not difficult at all, (2) somewhat difficult, (3) very difficult, and (4) extremely difficult.

## Discussion

### Principal Findings

After COVID-19 was declared a pandemic, quarantine and social distancing protocols were implemented to help mitigate disease spread in Texas. The implementation of these protocols was accompanied by several adverse effects on quality of life, which were independent of the SARS-CoV-2 infection. With the requirement for more individuals to stay at home and in isolation from friends, family, and colleagues, physical and mental health can depreciate. Mental health was drastically affected in our respondents owing to social isolation measures implemented by local municipalities in Texas. Overall, our survey respondents reported feeling more stress, anxiety, and sadness after COVID-19 was declared a pandemic. However, the long-term implications of quarantine protocols on mental health remain unknown. However, evidence from natural disasters suggest that mental health symptoms peak in the following months and can persist for years [8]. For example, 5% of the population of Texas affected by hurricane Ike met the criteria for major depressive disorder in the months following the storm [9]. Therefore, while a significant impact is demonstratable approximately 4 months into the quarantining protocol, there is potential for even greater impacts in the subsequent months.

Quarantine protocols can have a serious impact on physical health, leading to the development and progression of chronic diseases [10]. The American College of Sports Medicine recommends that most adults engage in moderate-intensity exercise training for more than 30 minutes for more than 5 days a week [11]. Contrary to Tinson et al [2], participants with no pre-existing medical conditions increased their physical activity after COVID-19 was declared a pandemic, with more individuals meeting or exceeding the recommended physical activity guidelines of the American College of Sports Medicine [11]. In this study, physical activity levels slightly—but not significantly—decreased among participants with pre-existing medical conditions, which indicates a potential risk of decreased overall health, potentially resulting in the exacerbation of medical conditions. Given that the risk of COVID-19 is significantly higher in subgroups of the population, which have pre-existing medical conditions, such subgroups are expected to decrease the time they spend outside of home in fear of unintended exposure to the virus.

### Limitations

Our study is limited by the self-reported nature of our survey and the convenience and snowball sampling method used to recruit participants. Further, without the use of geocoding, including zip codes, we are limited in our ability to generalize our results to different regions in Texas. Finally, our survey was distributed only in English, although Spanish is also a dominant language in several areas of Texas.

### Conclusions

The quarantine protocols implemented in Texas led to significant levels of stress and anxiety in our survey respondents. Physical activity can be considered a coping mechanism to alleviate the psychological distress people may feel. Psychological distress experienced during the COVID-19 pandemic is likely to have long-term clinical implications.
